# Off pump implantation of artificial chordae to correct mitral regurgitation – early results

**DOI:** 10.1186/1749-8090-8-S1-O291

**Published:** 2013-09-11

**Authors:** K Rucinskas, V Janusausakas, D Zakarkaite, S Aidietiene, R Samalavicius, G Speziali, A Aidietis

**Affiliations:** 1 Department of Cardiovascular Medicine, Vilnius University, Vilnius, Lithuania; 2 Department of Cardiothoracic Surgery, University of Pittsburgh Physicians, Pittsburgh, PA, USA

## Background

New technologies for mitral valve repair are emerging. One of them is NeoChord DS1000 system (Neochord, Inc., Minneapolis, MN). This device is designed to deliver artificial chordae tendinae to mitral valve leaflets on a beating heart through left ventricle wall. This is our initial experience with this device.

## Methods

This data is part of multi-center, nonrandomized, prospective Transapical Artificial Chordae Tendinae [TACT] trial. 13 patients with severe mitral regurgitation caused by isolated posterior mitral valve leaflet prolapse, were included in the study. The operation was done through left anterolateral thoracotomy on a beating heart. With TEE guidance, the NeoChord DS1000 was introduced into the left ventricle. The prolapsing segment was grasped with the device and ePTFE suture was attached to the posterior leaflet. Six patients received 2 ePTFE sutures to achieve competence of mitral valve, four patients - 3 ePTFE sutures, and two patients - 4 ePTFE sutures. At six months follow up all patients had TTE.

## Results

Median duration of operation was 110 min (80 to 150 min). Less than second degree mitral regurgitation in six months was achieved in 11 (85%) patients. 1 (8%) patient has developed recurrent third degree mitral regurgitation in one month. 1 (8%) patient has been converted to conventional mitral valve repair during procedure. There were no other adverse events related to the procedure.

## Conclusions

Treatment of mitral regurgitation by off-pump implantation of artificial chordae using NeoChord device is feasible and safe method in selected group of patients. To evaluate long term results and improve patient selection more patients and later follow up data are needed.

## Trial registration

ClinicalTrials.gov ID: NCT01777815.

